# Supplementary material for: Cross-species cortical alignment identifies different types of anatomical reorganization in the primate temporal lobe
Source: eLife. 2020 Mar 23;9:e53232. doi: 10.7554/eLife.53232 (PMC7180052; doi:10.7554/eLife.53232)
Supplement: Supplementary file 1. — MSM configuration parameters for the registration of individual subject myelin maps prior to averaging to create a species myelin map. The parameters were kept constant for the three species and for both hemispheres. [file elife-53232-supp1.docx]

**Supplementary File 1**

| sulcal depth | myelin map |
| --- | --- |
| --simval=1,2,2,2  --sigma_in=4,4,2,1  --sigma_ref=2,2,1,1  --lambda=0,0.1,0.2,0.3  --it=50,3,3,3  --opt=AFFINE,DISCRETE,DISCRETE,DISCRETE  --CPgrid=0,2,3,4  --SGgrid=0,4,5,6  --datagrid=4,4,5,6  --IN  levels=4 | --simval=2,2,2  --sigma_in=10,5,3  --sigma_ref=10,5,3  --lambda=0.1,0.1,0.3  --it=10,10,10  --opt=DISCRETE,DISCRETE,DISCRETE  --CPgrid=2,3,4  --SGgrid=4,5,6  --datagrid=4,5,6  levels=3  (--trans: sulcal depth registration) |

Supplementary File 1 – Supplementary Table related to Methods. MSM configuration parameters for the registration of individual subject myelin maps prior to averaging to create a species myelin map. The parameters were kept constant for the three species and for both hemispheres.
