# Supplementary material for: Cross-species cortical alignment identifies different types of anatomical reorganization in the primate temporal lobe
Source: eLife. 2020 Mar 23;9:e53232. doi: 10.7554/eLife.53232 (PMC7180052; doi:10.7554/eLife.53232)
Supplement: Supplementary file 2. — MSM configuration parameters. Settings for the MSM command for registrations using the macaque data as input (left panel) and chimpanzee data as input (right panel). The described ‘step’ refers to the numbering in Appendix 1—figure 1A. Parameters were identical for the left and right hemisphere. [file elife-53232-supp2.docx]

**Supplementary File 2**

| macaque mesh as input | chimpanzee mesh as input |
| --- | --- |
| step: 4🡪5  macaque-to-chimpanzee: MT+ ROI  --sigma_in=25  --lambda=0.1  --it=10  --opt=DISCRETE  --CPgrid=1  --SGgrid=3  --regoption=3  --regexp=2  --dopt=HOCR  --VN  --triclique  --k_exponent=2  --bulkmod=1  --shearmod=0.2  level=1 | step: 4🡪5  chimpanzee-to-human: MT+ ROI  --sigma_in=20,15,5  --lambda=0.05,0.1,0.1  --it=10,10,10  --opt=DISCRETE,DISCRETE,DISCRETE  --CPgrid=2,3,4  --SGgrid=4,5,6  --regoption=3  --regexp=2  --dopt=HOCR  --VN  --triclique  --k_exponent=2  --bulkmod=1  --shearmod=0.2  level=3 |
| step: 7🡪8  macaque-to-chimpanzee: three ROIs  --sigma_in=25,15,5  --lambda=0.01,0.01,0.1  --it=10,10,10  --opt=DISCRETE,DISCRETE,DISCRETE  --CPgrid=1,2,3  --SGgrid=3,4,5  --regoption=3  --regexp=2  --dopt=HOCR  --VN  --triclique  --k_exponent=2  --bulkmod=1  --shearmod=0.1  level=3  (--trans: single ROI registration) | step: 7🡪8  chimpanzee-to-human: three ROIs  --sigma_in=25  --lambda=0.001  --it=10  --opt=DISCRETE  --CPgrid=2  --SGgrid=4  --regoption=3  --regexp=2  --dopt=HOCR  --VN  --triclique  --k_exponent=2  --bulkmod=1  --shearmod=0.2  level=1  (--trans: single ROI registration) |
| step: 9🡪10  macaque-to-chimpanzee: myelin map  --sigma_in=10,5,3  --lambda=0.1,0.1,0.1  --it=10,10,20  --opt=DISCRETE,DISCRETE,DISCRETE  --CPgrid=2,3,4  --SGgrid=4,5,6  --IN  --excl  --regoption=3  --regexp=2  --dopt=HOCR  --triclique  --k_exponent=2  --bulkmod=1  --shearmod=0.2  level=3  (--trans: three ROI registration) | step: 9🡪10  chimpanzee-to-human: myelin map  --sigma_in=25,10,5  --lambda=0.2,0.5,0.5  --it=10,10,10  --opt=DISCRETE,DISCRETE,DISCRETE  --CPgrid=2,3,4  --SGgrid=4,5,6  --regoption=3  --regexp=2  --dopt=HOCR  --triclique  --k_exponent=2  --bulkmod=1.6  --shearmod=0.1  level=3  (--trans: three ROI registration) |
| step: 9🡪10  macaque-to-human  (input: macaque myelin map transformed by applying the macaque-to-chimpanzee registration)  --sigma_in=25,10,5  --sigma_ref=25,10,5  --lambda=0.1,0.1,0.1  --it=10,10,3  --opt=DISCRETE,DISCRETE,DISCRETE  --CPgrid=2,3,4  --SGgrid=4,5,6  --datagrid=4,5,6  --IN  --regoption=3  --regexp=2  --dopt=HOCR  --triclique  --k_exponent=2  --bulkmod=1.6  --shearmod=0.1  level=3  (--trans: chimpanzee-to-human myelin map registration) |  |

Supplementary File 2 – Supplementary Table related to Methods ‘Myelin-based Surface Registration’. MSM configuration parameters. Settings for the MSM command for registrations using the macaque data as input (left panel) and chimpanzee data as input (right panel). The described ‘step’ refers to the numbering in Figure S1A. Parameters were identical for the left and right hemisphere.
