# Supplementary material for: Cross-species cortical alignment identifies different types of anatomical reorganization in the primate temporal lobe
Source: eLife. 2020 Mar 23;9:e53232. doi: 10.7554/eLife.53232 (PMC7180052; doi:10.7554/eLife.53232)
Supplement: Transparent reporting form [file elife-53232-transrepform.docx]

***eLife’s* transparent reporting form**

We encourage authors to provide detailed information *within their submission* to facilitate the interpretation and replication of experiments. Authors can upload supporting documentation to indicate the use of appropriate reporting guidelines for health-related research (see [EQUATOR Network](http://www.equator-network.org/%20)), life science research (see the [BioSharing Information Resource](https://biosharing.org/)), or the [ARRIVE guidelines](http://www.plosbiology.org/article/info:doi/10.1371/journal.pbio.1000412) for reporting work involving animal research. Where applicable, authors should refer to any relevant reporting standards documents in this form.

If you have any questions, please consult our Journal Policies and/or contact us: [editorial@elifesciences.org](mailto:editorial@elifesciences.org).

**Sample-size estimation**

- You should state whether an appropriate sample size was computed when the study was being designed
- You should state the statistical method of sample size computation and any required assumptions
- If no explicit power analysis was used, you should describe how you decided what sample (replicate) size (number) to use

Please outline where this information can be found within the submission (e.g., sections or figure legends), or explain why this information doesn’t apply to your submission:

For average species myelin maps we used data from 20 humans, 5 macaques, 26 chimpanzees. No sample-size calculation was performed. This sample size is thought to be sufficient to generate an average species myelin map with distinct features. For tractography, we used data from 20 humans, 5 macaques and 5 chimpanzees. Sample size in non-human primates was limited due to technical feasibility. The sample-sizes are reported in the `Material and Methods` section.

**Replicates**

- You should report how often each experiment was performed
- You should include a definition of biological versus technical replication
- The data obtained should be provided and sufficient information should be provided to indicate the number of independent biological and/or technical replicates
- If you encountered any outliers, you should describe how these were handled
- Criteria for exclusion/inclusion of data should be clearly stated
- High-throughput sequence data should be uploaded before submission, with a private link for reviewers provided (these are available from both GEO and ArrayExpress)

Please outline where this information can be found within the submission (e.g., sections or figure legends), or explain why this information doesn’t apply to your submission:

No task or experiment was performed. The average species myelin maps and tractography results replicate numerous previous reports from the literature. No data was excluded from the analysis.

**Statistical reporting**

- Statistical analysis methods should be described and justified
- Raw data should be presented in figures whenever informative to do so (typically when N per group is less than 10)
- For each experiment, you should identify the statistical tests used, exact values of N, definitions of center, methods of multiple test correction, and dispersion and precision measures (e.g., mean, median, SD, SEM, confidence intervals; and, for the major substantive results, a measure of effect size (e.g., Pearson's r, Cohen's d)
- Report exact p-values wherever possible alongside the summary statistics and 95% confidence intervals. These should be reported for all key questions and not only when the p-value is less than 0.05.

Please outline where this information can be found within the submission (e.g., sections or figure legends), or explain why this information doesn’t apply to your submission:

We compared tract extension ratios across tracts and species using a non-parametric permutation test implemented in PALM (Winkler et al. 2014). Details about the test are provided in the `Material and Methods – Predicted Tract Maps` section. The resulting measures are reported in the `Results` section and visualized in Figure 5 and Appendix 3 – Figure 5. Numerical data underlying the graphs and the statistical tests are provided as Source Data (Appendix3_Figure2_source_data.csv).

(For large datasets, or papers with a very large number of statistical tests, you may upload a single table file with tests, Ns, etc., with reference to sections in the manuscript.)

**Group allocation**

- Indicate how samples were allocated into experimental groups (in the case of clinical studies, please specify allocation to treatment method); if randomization was used, please also state if restricted randomization was applied
- Indicate if masking was used during group allocation, data collection and/or data analysis

Please outline where this information can be found within the submission (e.g., sections or figure legends), or explain why this information doesn’t apply to your submission:

There was no group allocation into experimental groups as the groups were different species.

**Additional data files (“source data”)**

- We encourage you to upload relevant additional data files, such as numerical data that are represented as a graph in a figure, or as a summary table
- Where provided, these should be in the most useful format, and they can be uploaded as “Source data” files linked to a main figure or table
- Include model definition files including the full list of parameters used
- Include code used for data analysis (e.g., R, MatLab)
- Avoid stating that data files are “available upon request”

Please indicate the figures or tables for which source data files have been provided:

Numerical data underlying the graphs in Figure 5 and Appendix3 – Figure 2 is provided in form of Source Data files. Upon acceptance, results scene files and processing code will be made available from the Wellcome Centre for Integrative Neuroimaging’s GitLab at git.fmrib.ox.ac.uk/neichert/project_MSM. The result workbench scene files contain group-level results which allow interactive inspection of the surface data. The full parameter list required to run the between-subject MSM registration to create the average myelin maps and to run the cross-species registration MSM are provided in the Supplementary File 1 and 2.
